# Frequency and Reasons for Referrals to Liaison Psychiatry in Acute Hospital Settings: A Multi-Site Service Evaluation Across GMMH NHS Foundation Trust

**DOI:** 10.1192/bjo.2026.11647

**Published:** 2026-06-30

**Authors:** Ahmed Ghaly

**Affiliations:** Greater Manchester Mental Health NHS Foundation Trust, Manchester, United Kingdom

## Abstract

**Aims:**

Liaison psychiatry plays a key role in managing mental health presentations within acute hospital settings. However, variation in referral patterns and service utilisation may impact effectiveness and resource planning. This service evaluation aimed to examine the frequency, reasons, demographics, referral sources, and outcomes of referrals to liaison psychiatry services across multiple acute hospitals within a large NHS mental health trust, and to identify potential gaps in current service provision.

**Methods:**

A retrospective observational service evaluation was conducted using routinely collected electronic health record data for all referrals to liaison psychiatry services across seven acute hospital sites over a twelve-month period. Data extracted included patient demographics (age, gender, ethnicity), referring departments, reasons for referral, and outcomes following assessment by liaison psychiatry teams. Descriptive analyses were performed to explore referral volumes, site-level variation, demographic patterns, and intervention outcomes.

**Results:**

A total of 32,209 referrals were analysed across the seven hospital sites. Emergency Departments accounted for the majority of referrals at all sites (approximately 67–85%). The most common reasons for referral were suicidal thoughts (42%), followed by low mood (23.4%) and self-harm/overdose (18.5%). Significant variation was observed in referral rates per population across sites, with some hospitals demonstrating substantially higher activity than others. Referrals predominantly involved working-age adults, while those under 20 years and older adults were proportionally lower at several sites. Ethnicity data revealed a predominantly White British referral population, though substantial missing data limited interpretation at some locations. The most frequent outcome was discharge with GP follow-up (38%), while smaller proportions were referred to community mental health teams, home treatment teams, or admitted to psychiatric wards.

**Conclusion:**

This multi-site service evaluation demonstrates that liaison psychiatry services across acute hospitals are predominantly accessed via Emergency Departments, with suicidal ideation and mood-related concerns representing the most common referral reasons. Marked variation in referral volumes, demographic representation, and outcomes across sites suggests differences in service utilisation, integration, and potentially unmet need. Data quality issues, particularly around ethnicity and outcome coding, were identified. These findings highlight opportunities for improving referral pathways, strengthening liaison integration beyond Emergency Departments, and enhancing data recording to support future service planning, workforce allocation, and targeted quality improvement initiatives.

